# Comparative transcriptomic analysis identifies distinct molecular signatures and regulatory networks of chondroclasts and osteoclasts

**DOI:** 10.1186/s13075-020-02259-z

**Published:** 2020-07-10

**Authors:** Nazir M. Khan, Kari B. Clifton, Joseph Lorenzo, Marc F. Hansen, Hicham Drissi

**Affiliations:** 1 Department of Orthopaedics, Emory University School of Medicine, Atlanta, GA-30033 USA; 2 Atlanta VA Medical Center, Decatur, GA USA; 3 Department of Biology, University of West Florida, Pensacola, FL USA; 4 Department of Medicine, UConn Health, Farmington, CT USA; 5 Department of Orthopaedic Surgery, UConn Health, Farmington, CT USA; 6 Center for Molecular Medicine, University of Connecticut Health Center, Farmington, CT USA

**Keywords:** Chondroclasts, Osteoclasts, Gene arrays, Transcriptomics, Regulatory network, Fracture callus

## Abstract

**Background:**

Chondroclasts and osteoclasts have been previously identified as the cells capable of resorbing mineralized cartilage and bone matrices, respectively. While both cell types appear morphologically similar, contain comparable ultrastructural features, and express tartrate-resistant acid phosphatase (TRAP), however, no information is available about the genomic similarities and differences between osteoclasts and chondroclasts.

**Methods:**

To address this question, we laser captured homogeneous populations of TRAP-positive cells that interact with bone (osteoclasts) and TRAP-positive cells that interact with mineralized cartilage (chondroclasts) on the same plane from murine femoral fracture callus sections. We then performed a global transcriptome profiling of chondroclasts and osteoclasts by utilizing a mouse genome Agilent GE 4X44K V2 microarray platform. Multiple computational approaches and interaction networks were used to analyze the transcriptomic landscape of osteoclasts and chondroclasts.

**Results:**

Our systematic and comprehensive analyses using hierarchical clustering and principal component analysis (PCA) demonstrate that chondroclasts and osteoclasts are transcriptionally distinct cell populations and exhibit discrete transcriptomic signatures as revealed by multivariate analysis involving scatter plot, volcano plot, and heatmap analysis. TaqMan qPCR was used to validate the microarray results. Intriguingly, the functional enrichment and integrated network analyses revealed distinct Gene Ontology terms and molecular pathways specific to chondroclasts and osteoclasts and further suggest that subsets of metabolic genes were specific to chondroclasts. Protein-protein interaction (PPI) network analysis showed an abundance of structured networks of metabolic pathways, ATP synthesis, and proteasome pathways in chondroclasts. The regulatory network analysis using transcription factor-target gene network predicted a pool of genes including ETV6, SIRT1, and ATF1 as chondroclast-specific gene signature.

**Conclusions:**

Our study provides an important genetic resource for further exploration of chondroclast function in vivo. To our knowledge, this is the first demonstration of genetic landscape of osteoclasts from chondroclasts identifying unique molecular signatures, functional clustering, and interaction network.

## Background

The skeleton is a specialized and dynamic organ that undergoes continuous regeneration and remodeling. The process of bone modeling is responsible for the formation and maintenance of the shape of bone [1]. The cells that are responsible for bone remodeling are the osteoclasts, which are originated from the fusion of hematopoietic cells of the monocyte lineage [2]. The osteoclasts are central mediators of both normal bone remodeling and pathologies associated with excessive bone resorption [3]. The mature osteoclast is a large multinucleated, highly polarized cell with a convoluted plasma membrane region called the ruffled border, which facilitate the efficient resorption of bone [4]. During this process, osteoclasts firmly attach to bone surface, polarize their structure, seal the resorption compartment from the extracellular space, and secrete acid and a variety of degradative enzymes into the resorption compartment.

In addition to the osteoclasts, chondroclasts form on calcified cartilage to resorb mineralized cartilage matrix during endochondral ossification [5]. The majority of our knowledge about chondroclasts comes from ultrastructural analyses [6, 7]. Chondroclasts appear to be similar to osteoclasts in that they are multinucleated; contain similar ultrastructural features such as similar nuclear profiles, abundant mitochondrial profiles, and intracytoplasmic vesicles; and express tartrate-resistant acid phosphatase (TRAP) which are mainly secreted in the ruffled border area of both chondroclasts and osteoclasts [5]. Specific differences include a decreased size of the ruffled border and clear zone as well as increased accumulation of TRAP in chondroclasts relative to osteoclasts. Evidence that osteoclasts and chondroclasts stem from the same myeloid progenitors came from genetic mouse models in which Receptor Activator of NF-κB (RANK) or RANK-ligand (RANKL) were abrogated. In these mutants, neither osteoclasts nor chondroclasts form [8, 9]. Likewise, it has been demonstrated that RANKL is expressed at sites of osteoclast and chondroclast formation [10]. This hypothesis is further supported by the finding that osteopetrotic humans also have defects in both chondroclasts and osteoclasts [11].

The previous studies provided limited evidence that osteoclasts and chondroclasts are identical, except in their substrate. The other study has suggested that there may be differential expression of matrix metalloproteinases (MMP) in chondrocytes and osteoclasts [12]. However, these studies have almost been limited to only morphological, ultrastructural, and rudimentary expression analysis and have therefore not addressed the genome-wide transcriptomic landscape and molecular differences between osteoclasts and chondroclasts. There exists a significant gap in knowledge about how nearby substrates (the bone or calcified cartilage) regulate their function and behavior of osteoclasts and chondroclasts. We hypothesize that the adjacent substrate near osteoclasts and chondroclasts influences the expression levels of genes implicated in a variety of regulatory pathways and biological processes and hence represents transcriptionally distinct population. Therefore, a genome-wide profiling for transcriptional changes is required to overview the molecular processes and pathways and identifying set of putative gene signature specific to osteoclasts and chondroclasts.

In this study, we performed global transcriptome profiling of chondroclasts and osteoclasts by utilizing a mouse genome Agilent GE 4X44K V2 microarray platform. The systemic and comprehensive transcriptome analyses aid in our understanding of genetic diversity and molecular functionality of osteoclasts and chondroclasts in vivo. Additionally, the functional enrichment and integrated network analyses revealed distinct Gene Ontology terms and molecular pathways specific to chondroclasts and osetoclasts. The regulatory network analysis using transcription factor-target gene network predicts a pool of genes including ETV6, SIRT1, and ATF1 as chondroclast-specific gene signature. Our study provides an important genetic resource for further exploration of chondroclast function in vivo. To our knowledge, this is the first demonstration of unique molecular signatures that differentiate osteoclasts from chondroclasts.

## Methods

### Fracture model for generation of TRAP-positive cells

A closed femoral fracture model was used to generate an early-stage callus containing both calcified cartilage matrix and bony callus. Twelve-week-old male C57BL/6 J mice (stock no: 000664, Jackson Laboratory, Bar Harbor, ME) were used. Animals were given Buprenorphine SR (0.1 mg/kg body weight) as analgesia by subcutaneous injection prior to the procedure, and once a day for 3 days following the fracture in accordance with a protocol approved by the Animal Care Committee at UCHC (University of Connecticut Health Center, Farmington, CT). Mice were anesthetized with 4% isoflurane prior to the procedure, and left leg was shaved and cleaned with 70% ethanol. A hole was made in the femoral intramedullary canal at the intracondylar notch using a 25-gauge needle, and a 0.015-in.-diameter stainless steel pin was inserted into the intramedullary canal to stabilize the left femur prior to fracture. A closed mid-diaphyseal femoral fracture was produced by a 3-point bending fracture device [13, 14]. The limbs were radiographed (Faxitron, Wheeling, IL) immediately after fracture to ensure proper pin placement and subsequently to verify fracture. Mice were euthanized on days 12 post-fracture by CO_2_ asphyxiation followed by cervical dislocation.

### Sectioning and staining

Femurs were harvested 12 days post-fracture, cleaned of soft tissue, and fixed in 10% formalin. Femurs were subsequently decalcified in EDTA, dehydrated through successive grades of ethanol and xylene, and paraffin embedded. Sequential sections (7 μm) were cut in the frontal plane and stained for TRAP-positive cells using the K-ASSAY TRAP Staining Kit (Kamiya Biomedical, Seattle, WA). After staining, sections were dehydrated through ascending grades of ethanol, submerged in xylene for 30 s, and then rapidly dried with forced air.

### Laser capture microdissection and RNA isolation

Laser capture microdissection was performed with a PixCell II laser capture system (Life Technologies, Carlsbad, CA) under conditions described previously [15]. A laser spot size of 7.5 μm diameter was used, with 100 mW power and 3.4 ms pulse duration. Approximately 50 individual cells (chondroclasts or osteoclasts) were captured per cap, and each capture was performed in triplicate using sections from three different mice. Osteoclasts and chondroclasts were visualized by TRAP staining and further distinguished from each other by counterstaining with Alcian Blue, a specific stain for cartilage. The TRAP-positive cells adjacent to the mineralizing cartilage were chondroclasts while those adjacent to the mineralizing bone are osteoclasts. Individual TRAP-positive cells from the external callus were observed and identified under × 100 magnification and captured on the Arcturus CapSure™ Macro LCM Caps (Life Technologies, Carlsbad, CA). Individual caps were examined, and debris or excess material was removed by gently touching the cap to the adhesive strip on a Post-It™. The caps were inserted into Eppendorf tubes, where the cells were lysed by overnight digestion with Proteinase K at 37 °C. Total RNA was isolated from chondroclasts or osteoclasts with the Arcturus Paradise™ Extraction and Isolation Kit (Life Technologies, Carlsbad, CA) following the manufacturer’s instructions. RNA concentration was measured with a NanoDrop™ spectrophotometer.

### Microarray analyses

RNA was sent to GenUs BioSystems (Northbrook, IL) for microarray analysis. The concentration and quality of total RNA were assessed using an Agilent Bioanalyzer with the Agilent RNA6000 Pico Lab Chip (Supplemental Figure 1). Labeled cRNA was prepared from approximately 20 ng of total RNA. Briefly, the Poly(A)+ RNA population within total RNA was amplified using MessageAMP II reagents (Life Technologies, Carlsbad, CA). After a second round of reverse transcription, second-strand cDNA synthesis, and purification of double-stranded cDNA, in vitro transcription was performed using T7 RNA polymerase in the presence of Biotin-11-UTP. The quantity and quality of the cRNA were assayed by spectrophotometry and on the Agilent Bioanalyzer. Two micrograms of purified cRNA was fragmented to uniform size and applied to Mouse GE 4X44k v2 Microarrays (Agilent Technologies, Design ID 026655) in hybridization buffer. Arrays were hybridized at 65 °C for 17 h in a rotating incubator and washed at 37 °C for 1 min. After staining with Streptavidin-Alexa555, rinsed and dried arrays were scanned with an Agilent G2565 Microarray Scanner (Agilent Technologies, Santa Clara, CA) at 5 μm resolution. Agilent Feature Extraction software was used to process the scanned images from arrays (gridding and feature intensity extraction), and the data generated for each probe on the array was analyzed with GeneSpring GX software (Agilent Technologies, Santa Clara, CA). To compare individual expression values across arrays, raw intensity data from each gene was normalized to the 75th percentile intensity of each array. Only genes with values greater than background intensity in all three replicates of at least one condition were used for further analysis.

### Differential gene expression analysis

Raw hybridization data were normalized using quantile normalization using the software package Bioconductor by Affymetrix (Affymetrix Inc.; Santa Clara, CA). The data were then analyzed using the GeneSpring GX7.3 (Agilent Technologies) and BeadStudio software (Illumina) packages for statistical analysis. A multiple testing correction test was applied to the analysis to reduce the unwanted variation inherent to the samples, and differentially expressed genes were identified using a two-sample *t* test using false discovery rate (FDR) for multiple test correction. Log fold change (logFC) > 2, and FDR *P*  < 0 .05 was set for statistically significant DEGs (differentially expressed genes). The heatmap for expression profiling of differentially expressed genes was generated by R package of pheatmap. Additionally, unsupervised hierarchical clustering analysis was done using Euclidean clustering distance and average clustering method. Further, to identify the genetic overlap between chondroclasts and osteoclasts, we performed principal component analysis (PCA) based on the expression profiles of differentially expressed genes using the ClustVis [16].

### Quantitative PCR analysis

The linear amplified RNA (aRNA) generated for microarray analysis was used as template for cDNA synthesis for use in gene-specific quantitative PCR (qPCR) analysis. A total of 4 μg of each aRNA was reverse transcribed in reactions consisting of random hexamers, MMLV reverse transcriptase and buffer (Promega Corporation, Madison, WI), and dNTPs. The aRNA and random hexamers were initially heated to 70 °C for denaturation and cooled to 4 °C to allow for primer annealing before adding enzyme. Reactions were run using a BioRad MyCycler thermocycler and the program (37 °C/2 min, 42 °C/1 min, 50 °C/1 s) for 40 cycles, then 85 °C/5 min, and 4 °C hold. A single qPCR master mix was prepared for each sample consisting of the 4-μg equivalent cDNA obtained from RT reactions and Applied Biosystems Universal PCR Master Mix (Life Technologies, Grand Island, NY). Ten-microliter qPCR reactions consisting of approximately 20 ng of cDNA equivalent were run using an Applied Biosystems 7500 Real-Time PCR System and Applied Biosystems Custom Taqman 96-well qPCR arrays (Life Technologies, Grand Island, NY), containing assays for genes selected from the microarray data as well as endogenous controls and lineage-specific markers, and using the program (50 °C/2 min, 95 °C/10 min) followed by (95 °C/15 s, 60 °C/1 min) for 40 cycles. Data obtained was normalized for examining relative expression levels via quantitative ∆∆C_T_ analysis [17] with the mean C_T_ values of the collective endogenous controls used for internal normalization.

### Gene Ontology and pathway enrichment analyses

The differentially expressed genes between chondroclasts and osetoclasts were analyzed for functional enrichment of GO (Gene Ontology) terms and the Kyoto Encyclopedia of Genes and Genomes (KEGG) pathways using STRING (Search Tool for the Retrieval of Interacting Genes), a network cluster tool [18]. Functional enrichment was performed in two categories of GO terms: biological process (BP) and molecular function (MF). All genes in the genome were used as the enrichment background. GO terms with a *P* value of < 0.05, a minimum count of 3, and a gene term ratio of > 3% (the term ratio is calculated as ratio of the number of hit genes within a particular category to the total number of genes reported in same category) were collected. The significance of enriched pathways and *P* values was calculated based on the cumulative hypergeometric *t* test, and false discovery rate (FDR) was used for multiple correction testing. The significantly enriched GO terms for MF and BP in chondroclasts and osteoclasts were represented by “advanced bubble chart” demonstrating the number and significance of differentially expressed genes enriched in the pathway. KEGG pathways enriched in chondroclasts were shown as “Circos plot,” which represents the selected significantly enriched pathways and associated with genes in chondroclasts.

### Integrated protein network analysis

We also analyzed the functional interactions among proteins to reveal the biological significance of enriched pathways and associated genes using integrated network analysis by STRING (version 11.0) [18]. The differentially expressed genes between osteoclasts and chondroclasts were used as input gene set, and protein-protein interactions (PPIs) were analyzed for experimentally validated interactions with a combined score of > 0.7 indicating high confidence score for significant interaction. The interaction network was visualized by Cytoscape (version 3.7.1) [19, 20], and CytoNCA plugin (version 2.1.6) was used to analyze the topological properties of nodes in the PPI network [21]. To identify the most significant nodes, the connectivity degree of networks was assessed and significance was calculated using score ranking of each node. The proteins with a degree centrality of > 5.0 were identified as hub proteins.

### Network cluster analysis

We performed network cluster analysis to identify the clustering modules in the PPI network of genes enriched in chondroclasts as compared to osteoclasts, using the Molecular Complex Detection Algorithm (MCODE) plugin (version 1.5.1) in Cytoscape [22]. The following criteria were used to identify the significant modules: “Degree cutoff=2,” “node score cutoff=0.2,” “Haircut=true,” “Fluff=false,” “k-core=2,” and “max depth=100.” The threshold score of 9 was chosen; therefore, the first three clusters (cluster 1, cluster 2, and cluster 3) with a clustering score of 21.01, 14, and 9 were selected and visualized with Cytoscape (version 3.7.1). The clustering modules having high node scores and connectivity degrees were considered as biologically significant clusters. The genes in the network cluster 1 were subjected to transcription factor (TF)-target gene regulatory network analysis to identify the potential genes signature specific to chondroclasts.

### Prediction of regulatory networks of transcription factors

We identified the candidate transcription factors to predict the coordinated regulation of genes involved in network cluster 1. We examined transcription factor binding motifs, enriched in the genomic regions of a query gene set using the iRegulon plugin (version 1.3) in Cytoscape [23]. The criteria set for motif enrichment analysis were as follows: identity between orthologous genes ≥ 0.0, FDR on motif similarity ≤ 0.001, and TF motifs with normalized enrichment score (NES) > 3. The ranking option for motif collection was set to 10 K (9713 PWMs), and a putative regulatory region of 20 kb centered around TSS (7 species) was selected for the analysis. Thereafter, TF-target pairs were obtained based on the TRANSFAC and JASPAR databases included in iRegulon plugin. Our analysis yielded 205 significantly enriched motifs (normalized enrichment score NES > 3) that clustered into 44 groups by similarity. More than 100 transcription factors were predicted to potentially bind the motifs enriched in network cluster 1 of chondroclast signature genes as compared to osteoclasts.

## Results

### Laser capture of the TRAP-positive cells, microarray analysis of osteoclasts and chondroclasts

**Fig. 1:**
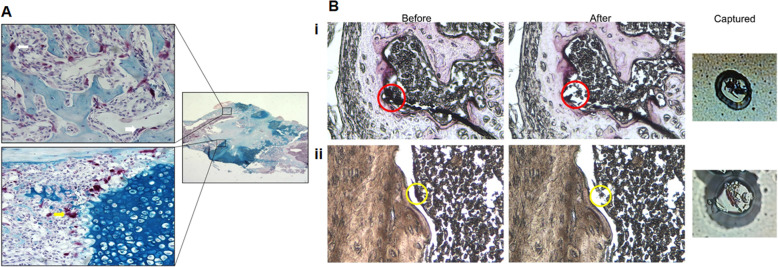
Localization of TRAP-positive chondroclasts and osteoclasts and LCM capture. **a** A mouse femur 12 days post-fracture stained with tartrate-resistant acid phosphatase (TRAP) (red) and counterstained with Alcian Blue, a specific stain for cartilage, shows TRAP-positive cells present in both the mineralizing cartilage (yellow arrow) and the mineralizing bone (white arrow) of the fracture callus. The TRAP-positive cells adjacent to the mineralizing cartilage are chondroclasts while those adjacent to the mineralizing bone are osteoclasts. **b** Seven-micrometer-thick sections cut in the frontal plane from demineralized femurs harvested 12 days post-fracture were stained for TRAP-positive cells and serially dehydrated in ascending grades of ethanol and finally xylene prior to laser capture microdissection. (i) TRAP-positive cells adjacent to mineralized cartilage (chondroclasts—red circles) are shown before LCM and after LCM, and the captured cells are shown attached to the LCM membrane. (ii) TRAP-positive cells adjacent to mineralized bone (osteoclasts—yellow circles) are shown before and after LCM, and the captured cells are shown attached to the LCM membrane

In order to identify chondroclasts and osteoclasts in the same tissue structure, we performed fracture experiments using 12-week-old male mice. During fracture healing, series of molecular events take place in sequential manner; however, there exists a significant temporal overlap in these cascades, which represent a continuum of changing cell populations and signaling events within the regenerating tissue. In murine femoral fracture, chondroclast activity was noted in fracture callus between days 9 and 15, whereas osteoclast was abundantly seen between days 11 and 20 post-fracture. Therefore, to isolate osteoclast and chondroclast in similar temporal and frontal plane from histological section of fracture callus, we chose day 12 post-fracture. Osteoclasts and chondroclasts on histological sections of fracture calluses were visualized by TRAP staining and distinguished from each other by counterstaining with Alcian Blue, a specific stain for cartilage (Fig. 1a). The TRAP-positive cells adjacent to the mineralizing cartilage were chondroclasts while those adjacent to the mineralizing bone are osteoclasts (Fig. 1b). There were a number of TRAP-positive cells that were not associated with either cartilage or bone; we did not capture these cells as it was unclear whether these would be chondroclasts or osteoclasts. RNAs were extracted from the laser-captured material isolated from fracture calluses of three independent animals and were subjected to microarray analyses. Quality and quantity of total RNA were assessed using an Agilent Bioanalyzer (Supplementary Figure 1). The average size of the RNA fragments was between 100 and 150 bp, though the majority was within the 150 bp.

### Chondroclasts and osteoclasts are transcriptionally distinct

**Fig. 2:**
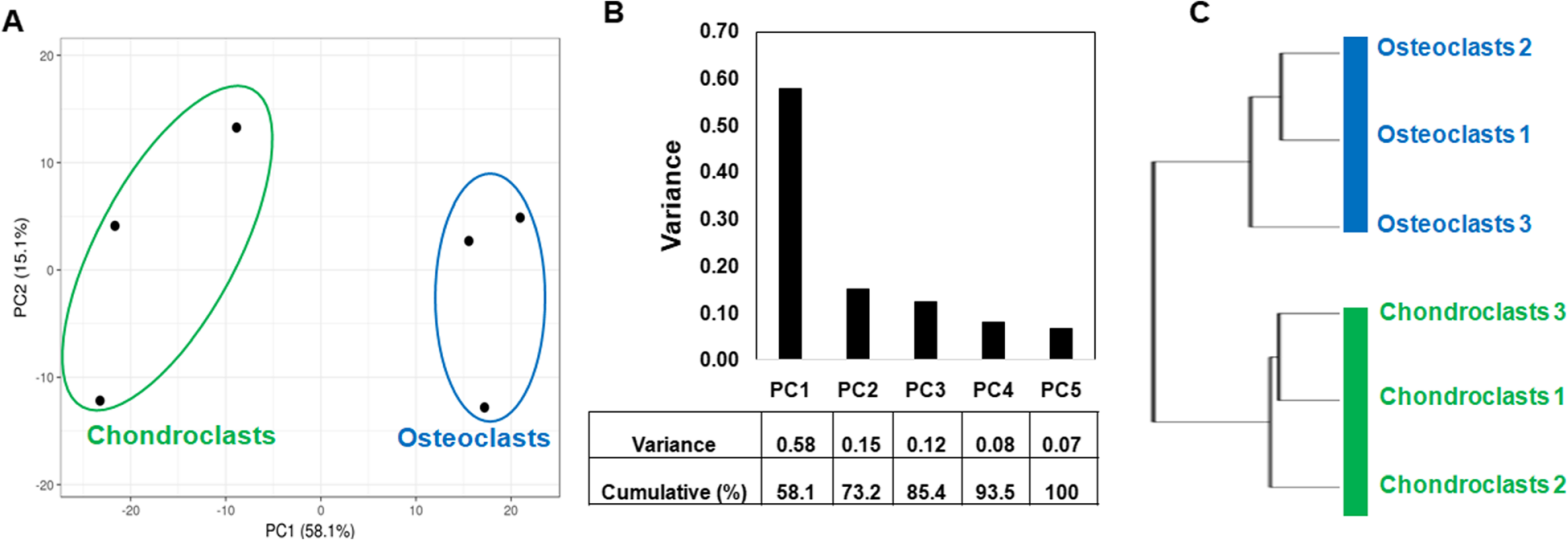
Chondroclasts and osteoclasts are transcriptionally distinct. Microarray analysis of RNA isolated from laser-captured chondroclasts and osteoclasts. Gene expression profiling for differentially expressed genes was compared in a multivariate analysis via ClustVis using hierarchical clustering and principal component analysis. The expression analysis led to the successful segregation of these cell populations by chondroclasts vs osteoclasts via principal component analysis (**a, b**) and hierarchical clustering analysis (**c**). **b** The PCA plot shows PC1 and PC2 indicating 58.1% and 15% of the total variance, respectively

Expression microarray was performed on a murine whole genome Agilent platform to investigate the global changes in gene expression of laser-captured osteoclasts and chondroclasts derived from fracture callus of C57BL6 mice. Normalized expression ratios and *P* values were calculated for all datasets using GeneSpring GX7.3. A total of 39,433 microarray probes that encompass 33,245 genes were used for analysis. After elimination of redundant probes, a total 2999 microarray probes showed significant expression (*P* < 0.05); however, 2413 probes (representing 1995 annotated genes) met the combined threshold criteria for differential expression (FC > 1.5 or < 0.667, and *P* < 0.05). There was a robust expression of TRAP gene (ACP5) in both cell populations. The established gene markers of osteoclasts (ATP6V0D2, CTSK, MMP9, SPP1, and CALCR) were strongly expressed in osteoclasts. We employed principal component analysis (PCA) to examine variation in transcript abundance among the genes contained in each array dataset. The reduction of this highly multidimensional dataset into two dimensions, or principle components, enables the unbiased comparison and visualization of total transcriptional activity between samples. The results suggest that the transcriptome profiles of osteoclasts and chondroclasts completely differed from each other (Fig. 2a). Furthermore, the variation captured in the first two principal components (PC1 and PC2) demonstrates broad differences between osteoclasts and chondroclasts, which accounted for total 73% of the variance in the dataset, suggesting a high degree of heterogeneity in global transcriptional activity among these samples (Fig. 2b). We next performed hierarchical clustering analysis based on global transcriptome profiling to assess the relationship, and consistent with PCA, our resulting dendrogram clearly distinguishes the osteoclasts from chondroclasts (Fig. 2c). These data suggest that chondroclasts and osteoclasts are transcriptionally distinct and thus provide a high level of confidence in further analyzing the gene expression patterns between these two populations. The cluster of chondroclasts as shown in Fig. 2a appears relatively wider; however, it is completely distinct from osteoclast cluster indicating the unique transcriptomic profile. The wider cluster pattern of chondroclasts and osteoclasts could be attributed to relatively lower number of experimental animals (*n* = 3), variation in metabolic and energetic state of chondroclast/osteoclast from different animals and the degree of inflammatory insult caused during femoral fracture, and also the extent of repair process at the time of harvest of chondroclast and osteoclast.

### Differential gene analyses reveal discrete transcriptomic signature of chondroclasts and osteoclasts

**Fig. 3:**
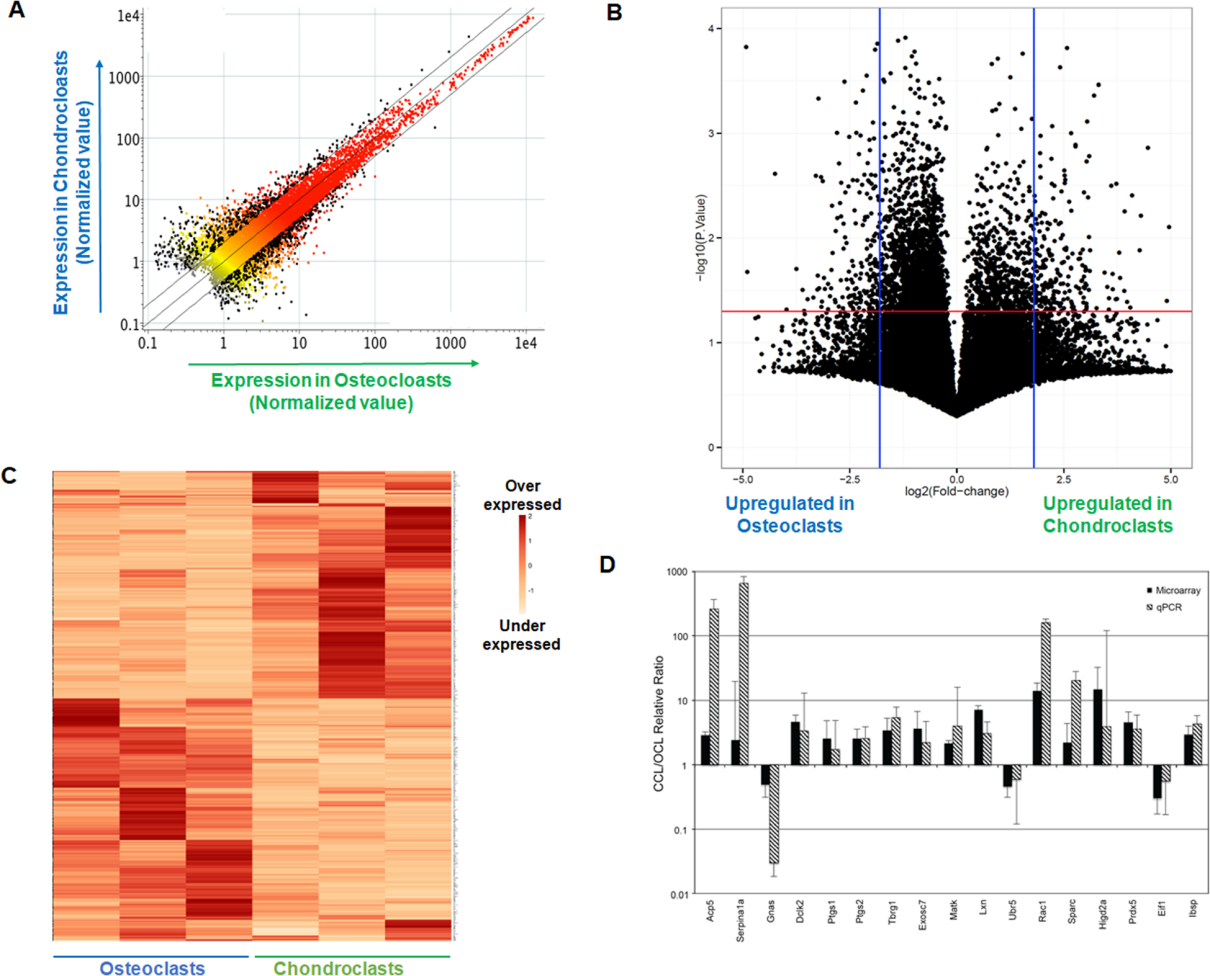
Differential gene analyses reveal discrete transcriptomic signature of chondroclasts and osteoclasts. The raw hybridization data were normalized using quantile normalization to the 75th percentile, and genes with differential expression levels greater than 2-fold (FDR *P* value < 0.05) were subjected to scatter plot (**a**), volcano plot (**b**), and heatmap analysis (**c**). There is a clear dichotomy between the genomic signatures of the chondroclasts and osteoclasts. **d** Validation of microarray results by quantitative PCR. Select genes were chosen for further analysis and validation of the microarray hybridization results. RNAs were reisolated from chondroclasts and osteoclasts captured by LCM from femurs harvested 12 days post-fracture and analyzed by qPCR for expression of genes. The results compare the levels of expression of the qPCR and the microarray hybridization

Genome-wide expression profiles were compared between chondroclasts and osteoclasts to identify the differentially expressed genes between these cell types. Out of the whole mouse genome, 9576 genes were detected above background in both cell types. A total of 851 microarray probes representing 732 annotated genes (FDR *P* < 0.05, FC > 2.0) were significantly different between the chondroclasts and osteoclasts (Fig. 3a). The Pearson correlation was also used to test similarities in the patterns of gene expression, and data as shown by scatter plot and volcano plot revealed that out of 732 differentially expressed genes, 375 were upregulated in chondroclasts and 357 genes were upregulated in osteoclasts (Fig. 3a, b). A heatmap analysis revealed differential expression of these genes, which discriminate well between chondroclasts and osteoclasts (Fig. 3c). The expression levels of P2rx5, Nxn, Gspt1, Serp1, Scrn1, and Rac1 were significantly higher in chondroclasts, whereas expression of Psmb9, Dot1l, Wasf2, and Aldh3a1 were significantly higher in osteoclast samples (Fig. 3c). We next performed quantitative real-time PCR to validate a subset of genes that were differentially expressed in osteoclasts and chondroclasts. To this end, we designed custom-made ABI low-density qPCR arrays to validate the changes in gene expression. We selected genes that exhibited more than 2-fold differential expression between chondroclasts and osteoclasts. Our qPCR analysis as shown in Fig. 3d demonstrated the patterns of several genes including Rac1 (a GTPase related to Ras superfamily) and Serp1 (a serine peptidase inhibitor) which were upregulated in chondroclasts over osteoclasts as well as Eif1 (a translation initiation factor), GNAS (a guanine nucleotide binding protein), and Ubr5 (an E3 ubiquitin ligase component) which were downregulated in chondroclasts relative to osteoclasts (Fig. 3d). We also validated the microarray results by examining the expressions of a subset of proteins such as Lmp2, Psat1, Ufsp2, Nxn, and Wnt5a using immunofluorescent staining in histological section of chondroclasts and osteoclasts (Supplementary Figure 2). TRAP staining was also performed to confirm the specificity of immunostaining in these cells, and nuclear staining was performed to visualize the nucleus. Altogether, these data suggest chondroclasts and osteoclasts exhibit unique transcriptomic signature, which are completely distinct from each other.

### Functional annotation clustering identifies subsets of metabolic genes specific to chondroclasts

**Fig. 4:**
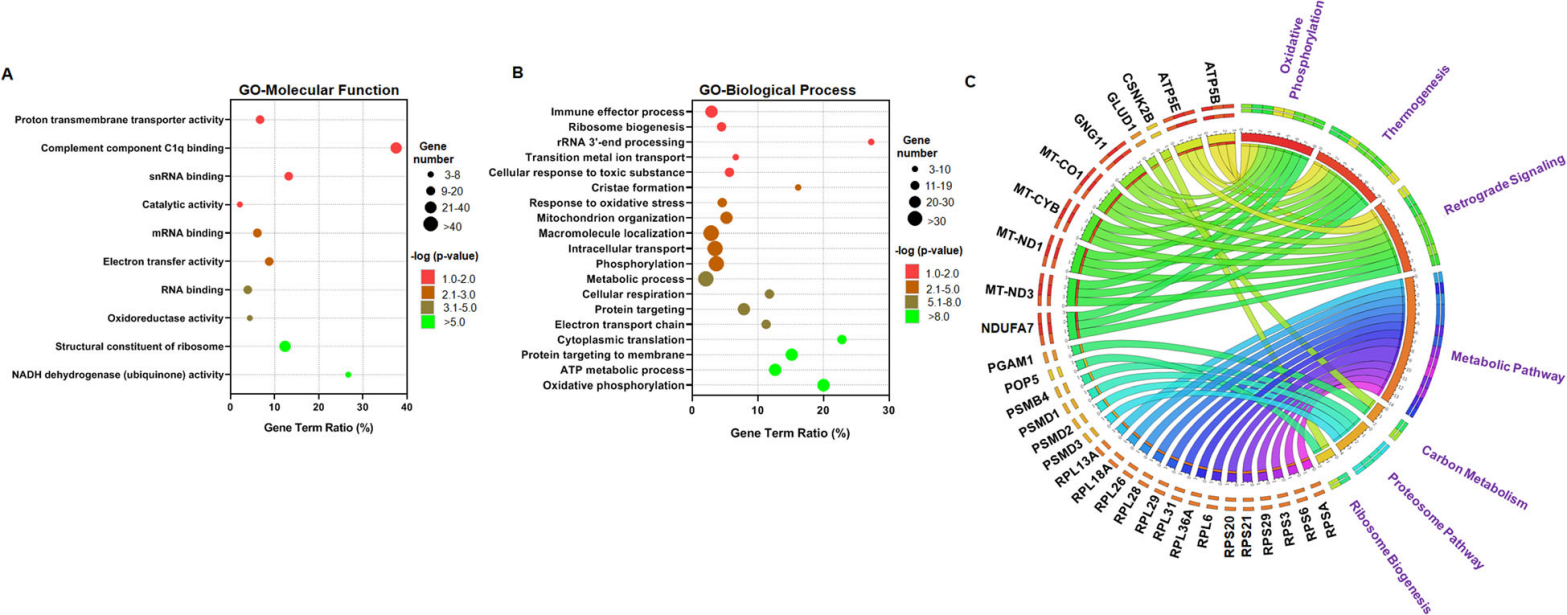
Functional annotation clustering identifies subsets of metabolic genes specific to chondroclasts. GO analysis of genes upregulated in chondroclasts for molecular function (**a**) and biological process (**b**). The top enrichment pathways were shown in the advance bubble chart. *Y*-axis label represents pathway, and *X*-axis label represents gene term ratio (gene term ratio = gene numbers annotated in this pathway term/all gene numbers annotated in this pathway term). Size of the bubble represents the number of genes enriched in the GO terms, and color showed the FDR *P* value of GO terms. **c** KEGG pathway enrichment analysis of chondroclast-specific genes was represented by Circos plot. The associated genes and KEGG pathways are indicated outside the circle

We next performed functional annotation clustering using GO and KEGG pathway analysis to reveal the biological processes and pathways associated with genes upregulated in chondroclasts. The GO enrichment analysis showed that the genes expressed in chondroclasts were involved in 12 GO terms for molecular function (MF) and 119 terms for biological process (BP). Our GO analysis for both molecular function and biological process showed that chondroclast-specific genes were enriched in metabolic process, transport activity, and cellular respiration including oxidoreductase and electron transport chain activity (Fig. 4a, b). These results suggest that cell signaling involved in cellular metabolic pathways, notably the catabolic pathways of energy metabolism and oxidative phosphorylation, was enriched in chondroclasts. We also examined the genes based on KEGG pathway enrichment analysis, and our data showed that “oxidative phosphorylation” is the most enriched pathway (Fig 4c), which supports the results of the GO analysis. The Circos plot analysis for the KEGG pathway showed that a large proportion (~ 10–18%) of chondroclast-specific genes were involved in energy metabolic pathways such as ribosome biogenesis, thermogenesis, oxidative phosphorylation, and the metabolic pathways, suggesting that signaling genes involved in these pathways such as PSMD2, ATP5B, MT-CO1, NDUFA7, GNG11, ATP5E, PSMB4, RPL31, GLUD1, and PGAM1 may constitute the signature genes of chondroclasts.

### Network analysis reveals the abundance of structured networks of metabolic pathways, ATP synthesis, and proteasome pathways in chondroclasts

We performed network analyses to identify functional relationships between subsets of genes that were significantly upregulated in chondroclasts. We found that the differentially expressed genes identified in our array data were integrated into functional networks by physical interactions or common signaling pathways involved in several essential energy metabolic processes, including ATP synthesis and electron transport chain. Network analysis of the integrated gene interaction network revealed 0.485 clustering coefficient, 11 connected component, network diameter of 13, network radius of 1, 0.247 network centralization, 21,792 (74%) shortest paths, 8.33 average neighbors, characteristic path length of 4.13, 0.049 network density, and 1.056 network heterogeneity (Supplementary Figure 3). The topological properties of our resulting interaction network showed significant results involving topological coefficients, betweenness centrality, clustering coefficient, and closeness centrality (Supplementary Figure 3).

**Fig. 5:**
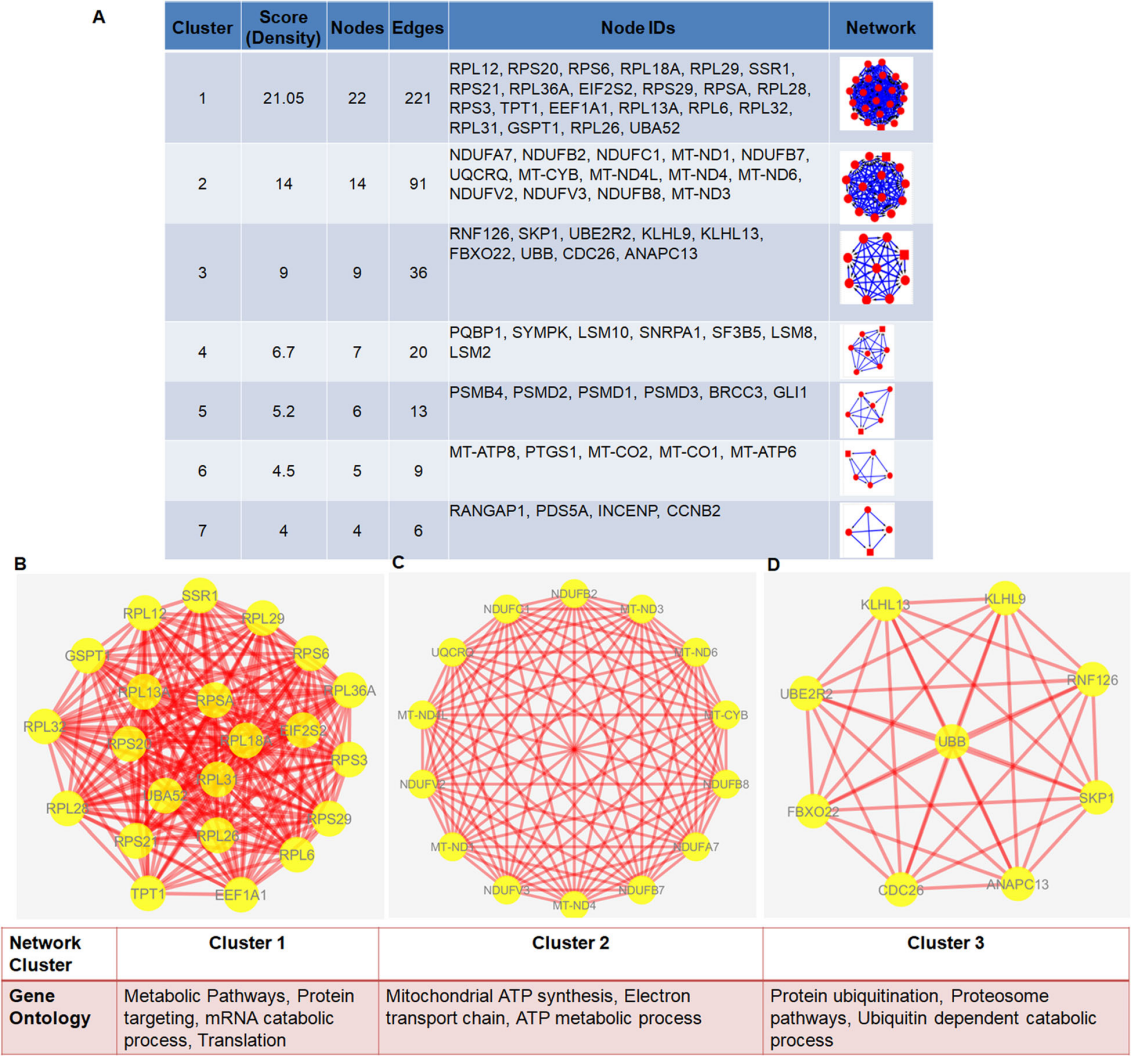
Network analysis reveals the abundance of structured networks of metabolic pathways, ATP synthesis, and proteasome pathways in chondroclasts. PPI network of genes upregulated in chondroclasts was constructed using STRING database utilizing experimentally validated interactions with a combined PPI score of > 0.7. Subnetwork analysis in the PPI network using MCODE identified the significant modules/clusters. **a** The cluster analysis in PPI network resulted in 10 clusters. Top 7 clusters were shown which include 67 nodes and 396 edges. Cluster 1 (**b**), cluster 2 (**c**), and cluster 3 (**d**) of top three network clusters in the subnetwork analysis of PPI network of IDD-related genes and enriched GO terms for biological processes were shown. The cluster networks were visualized by Cytoscape (version 3.7.1). All nodes are significantly enriched at FDR *P* value < 0.05

We next performed subnetwork analysis of the interaction network to identify significant clusters enriched in chondroclasts. Our MCODE cluster analysis resulted in 10 clusters that included 76 nodes and 405 edges (Fig. 5a). Our analysis revealed a total of 3 clusters with a score of ≥ 9. Cluster 1 exhibited the highest score, and 22 genes, such as RPL12, RPS20, RPS6, SSR1, RPS21, EIF2S2, RPS29, TPT1, EEF1A1, GSPT1, and UBA52, were included (Fig. 5b). Enrichment analysis demonstrated that the GO-BP terms enriched by these genes were associated with metabolic pathways, protein targeting, and translation (Fig. 5b). Genes in cluster 2 primarily included NDUFA7, MT-ND1, UQCRQ, MT-CYB, and MT-ND3, which were enriched in GO-BP terms associated with mitochondrial ATP synthesis and electron transport chain (Fig. 5c). Genes in cluster 3 included RNF126, SKP1, UBE2R2, KLHL9, KLHL13, FBXO22, UBB, CDC26, and ANAPC13, which were enriched in the proteasome pathways and ubiquitin-dependent catabolic pathways (Fig. 5d). Taken together, our interaction analysis revealed the abundance of structured networks of metabolic pathways, ATP synthesis, and proteasome pathways, indicating that metabolic signaling network in energy metabolism may be considered as chondroclast-specific signature gene network.

### Transcription factor-target gene regulatory network analysis predicts ETV6, SIRT1, and ATF1 as chondroclast signature transcription factors

**Fig. 6:**
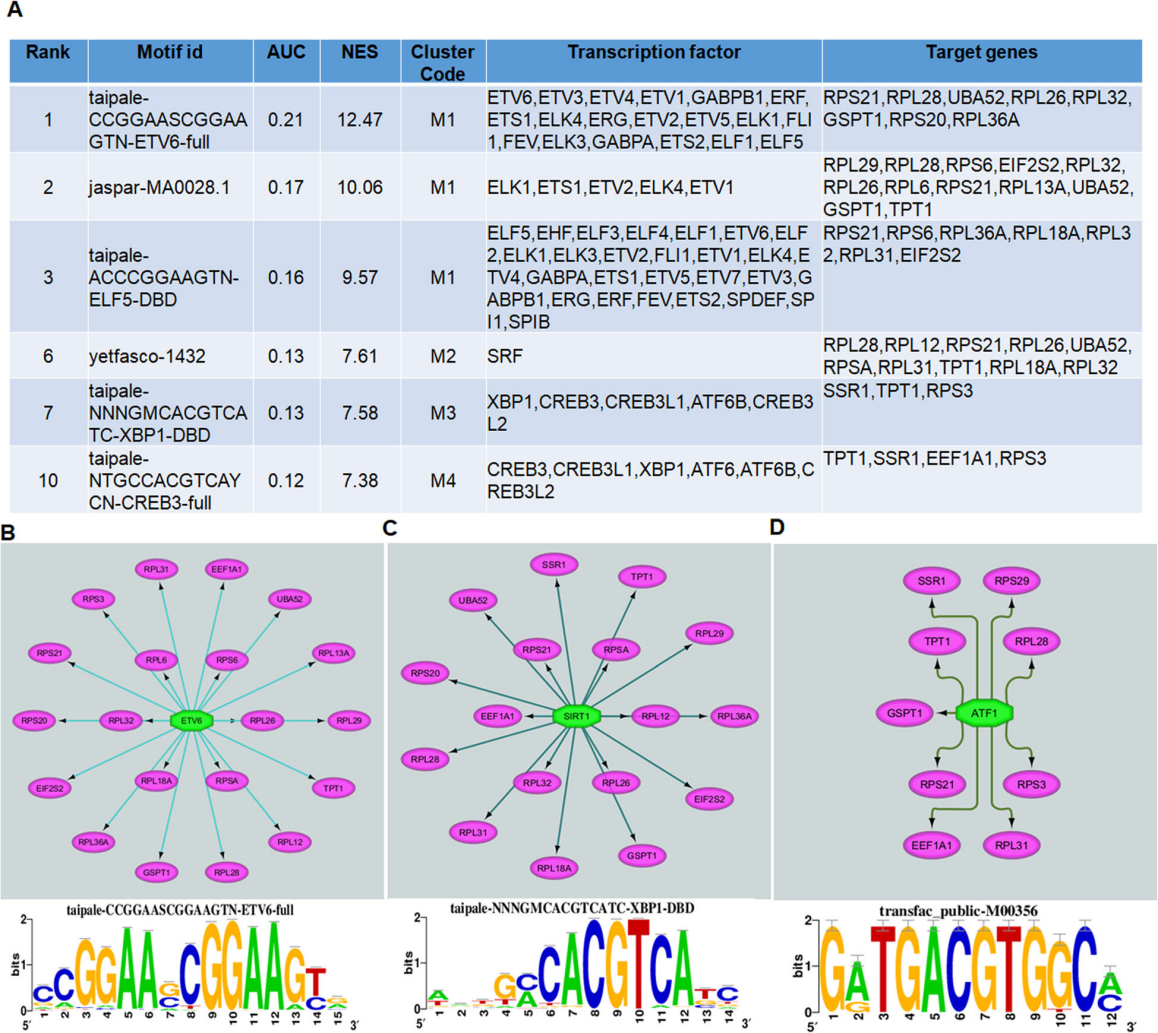
Transcription factor-target gene regulatory network analysis predicts ETV6, SIRT1, and ATF1 as chondroclast signature transcription factors. Transcription factor (TF)-gene target regulatory network analysis was performed in network cluster 1 using iRegulon plugin in Cytoscape (version 3.7.1). **a** The iRegulon analysis showed the top transcription binding motifs and their associated transcription factors enriched in the cis-regulatory regions of the twenty-two genes of network cluster 1. **b–d** Regulatory network analysis representing the predicted transcription factors (as octagonal nodes) of the target genes present in network cluster 1 (as oval nodes) showed ETV6 (**b**), SIRT1 (**c**), and ATF2 (**d**) as signature transcription factors of chondroclasts. The networks were visualized by Cytoscape (version 3.7.1), and transcription factors are significantly enriched at an adjusted *P* value < 0.05

We next determined the coordinated regulation of these metabolic signaling gene networks. To identify candidate transcription factors that regulate these genes, we performed transcription factor (TF)-gene target regulatory network analysis using iRegulon [23] to predict transcription factors that bind to the promoter of our gene query set. For this analysis, we chose to use cluster 1 because it was the most significant cluster from the above interaction network (Fig. 5a, b). Our analysis yielded 205 significantly enriched motifs (normalized enrichment score [NES] > 3) that clustered into 44 motif groups by similarity and 24 associated tracks. In total, > 100 transcription factors were predicted to potentially bind to the motifs present in genes involved in network cluster 1. To identify the best candidates from these transcription factors, several exclusion filters were applied including highest-ranking associated motif (> 7) and presence of number of targets (> 10), as well as maintenance of multiple motifs (> 4). These filters ended up having 6 motif IDs across 4 motif clusters (Fig. 6a). Motif cluster 1 included 19 transcription factors, whereas motif cluster 2 and 6 had only 5 and 1 transcription factor, respectively (Fig. 6a). Our analysis identified several novel candidate transcription factors, such as ETV6, SIRT1, ELK1, ATF1, SRF XBP1, and CREB3, that are involved in the transcriptional regulation of chondroclast-specific genes (present in cluster 1) (Fig. 6b–d). Moreover, the “regulator-target genes analysis” demonstrated that ETV6, SIRT1, and ATF1 potentially target a vast majority of the chondroclast signature genes as identified in cluster 1 (Fig. 6b–d). ETV6 (ETS Variant Transcription Factor 6) is required for hematopoiesis and maintenance of the developing vascular network, whereas SIRT1 (Sirtuin1) is a deacetylase which has been shown as a positive regulator of bone mass [24] and ATF1 is a cyclic AMP-dependent transcription factor which influences cellular physiologic processes by regulating the expression genes, which are involved in growth and survival. The predicted network identified specific interactions between regulators and target genes, including such targets of ETV6 as RPL13A, RPL31, RPS6, EIF2S2, and GSPT1 (Fig. 6b). Together, our results predict ETV6, SIRT1, and ATF1 as signature transcription factors for the chondroclasts.

## Discussion

The present study provides a comprehensive comparative transcriptomic profiling of osteoclasts and chondroclasts and has outlined detailed genetic landscape largely pointing towards functional clustering and interaction network. Our goal was to provide a broad compendium of gene expression differences between cartilage and bone resorbing cells based on their substrate locations, with a focus on genes involved in biological pathways and regulatory networks. Our data provide a significant and useful resource for the skeletal biologists. Our analyses identify significantly expanded cohorts of metabolic genes uniquely expressed by chondroclasts. These genes extend our understanding of chondroclasts as regulators of not only energy metabolism, but also ubiquitin-dependent catabolic pathways. Our analysis also points to an emerging understanding that chondroclasts express a variety of mitochondrial and ribosomal genes which enable them to adapt a metabolically active form. Our study suggests that chondroclasts function as a responder of cellular bioenergetics; however, further investigation of the role of identified metabolic genes on proliferation, differentiation, and function remains to be characterized. In addition to molecular characterization, the ultrastructural and morphological characterizations are also needed to define the structural aspect of chondroclasts. The detailed characterization of multinucleated chondroclast requires establishment of an in vitro culture condition and further investigation of presence of intracellular vacuoles, phagocytosed collagen fibers and lysosomal bodies, cytoplasmic extensions into cartilage, and presence or absence of specialized ruffled border structures similar to osteoclasts. However, for this process to be reproduced in vitro, it will require as yet undefined microenvironments. These might include different adjacent substrate or underlying microenvironment such as thin slices of mineralized and unmineralized articular cartilage or subchondral bony lesions in the culture condition. Further, series of assays need to be experimentally standardized to confirm the chondroclastic activity such as MMP13 activity in multinucleated chondroclast, expression of immunophenotypic markers characteristic of macrophages (CD68+, CD14+, and CD51−) and osteoclasts (CD68+, CD14−, and CD51+), presence of intracellular vacuoles, demonstrable acid phosphatase (TRAP+) activity, and glycosaminoglycan (GAG) release assay in the conditioned medium from resorbed cartilage.

Remodeling of mineralized cartilage is essential for the initiation of bone formation for endochondral ossification that occurs during skeletal development, homeostasis, and repair. Cells have been identified with the unique function of remodeling mineralized matrix. The term chondroclasts is increasingly assigned to multinucleated, TRAP-positive cells capable of resorbing mineralized cartilage matrix [3]. In general, our knowledge of differences between osteoclasts and chondroclasts is limited to ultrastructural phenotyping [5–7]. While the genes that are necessary for osteoclastogenesis and osteoclast function have been widely characterized [25], to date, no information is available about the genomic similarities and differences between osteoclasts and chondroclasts. Our study provides first evidence of genomic differences between osteoclasts and chondroclasts based on their specific interaction with two distinct matrices.

We used a well-established murine femoral fracture model [26–28] to isolate TRAP-positive cells that interact with cartilage matrix and TRAP-positive cells that interact with bone matrix on the same plane. This was evident at 12 days post-fracture when the soft callus has not yet fully transitioned into bony callus in fractured C57/BL6 mice [27–29]. The fact that both cell types were available on the same plane within distinct geographical locations discarded any possibility for stage-dependent gene expression at least from the developmental perspective. Thus, the fracture callus model was highly useful as opposed to the known developmental models of the growth plate and trabecular bone previously used to characterize the phenotypes of osteoclasts and chondroclasts [3, 30, 31]. Laser capture microdissection (LCM) enabled harvesting of homogeneous populations of TRAP-positive cells. This method has been widely used to isolate single cells from histological sections [15, 32, 33]. To date, no other isolation method has proven successful to distinguish between osteoclasts and chondroclasts. The fact that little information is available about the gene expression signature of chondroclasts also precluded the use of FACS analyses for this purpose. In vitro differentiation of bone marrow derived macrophage into chondroclasts using mineralized cartilage or subchondral bony lesions as underlying substrate or microenvironment may offer an alternative cellular model to study the chondroclast; however, it requires extensive optimization of culture condition. As inflammatory multipotential myeloid cells differentiate into osteoclasts, we speculate that a similar precursor in subchondral pathological lesions has the ability to differentiate into chondroclasts and resorbs the cartilage.

Our genome-wide array analyses revealed large set of genes differentially expressed between chondroclasts and osteoclasts. Although total expressed genes in these two cell populations were similar, the substantially higher numbers of genes in chondroclasts relative to osteoclasts (375 vs 357) suggest that the chondroclasts may have more elaborated functionalities. However, the molecular mechanism and signaling pathways that drive their distinct functionalities are unclear. Therefore, the Gene Ontology and KEGG analysis were applied to perform the pairwise comparisons of the osteoclasts and chondroclasts. Notably, the functional pathways that were significantly overrepresented in chondroclasts included metabolic process, transport activity, cellular respiratory enzymes such as oxidoreductase, and others involved in electron transport chain and oxidative phosphorylation. It was likely that chondroclasts in a mineralized cartilaginous callus, which have not yet become bony in its majority, would exhibit higher expression of genes related to metabolism. For the metabolic pathway PSMD2, ATP5B, MT-CO1, and GLUD1 genes were significantly upregulated in chondroclast suggesting that these metabolic genes are likely attributes of chondroclasts in mineralized cartilage matrix which allow them to maintain adequate bioenergetic and catabolic activity during cartilage resorption. These data also point to enhanced metabolic components in chondroclasts compared to osteoclasts and also suggest that nearby substrate influences distinct bioenergetic and metabolic environments in their resorbing cells. Since these chondroclasts are derived from fracture model which is a highly active and vibrant model system and involved series of anabolic and catabolic cascade processes, the enrichment of metabolic pathways as chondroclast signature genes could be attributed to the active and dynamic nature of our elected cellular model system for chondroclasts. The disruption to the normal bone microenvironments caused during fracture leads to the active interactions of cell populations from the medullary space, periosteum, and enveloping muscular tissues. Therefore, signaling and cellular contributions from these different tissues and their microenvironments may affect the genomic and epigenomic profile of studied chondroclasts and osteoclasts and may contribute to the enrichment of metabolically active genes. Whether this is general findings to chondroclast or influenced by energetic and vibrant nature of our chosen model system is further area of investigation and warrants future systematic transcriptomic studies in the culture condition of chondroclast in active and dormant stage.

Finally, the comprehensive view of the chondroclast transcriptome established energy metabolism as a main functional network of chondroclasts. Further, regulatory network analysis suggests that genes enriched in ATP synthesis and proteasome pathways and metabolic pathways are regulated by three main factors—ETV6, SIRT1, and ATF1. Although these data indicate that chondroclasts are endowed with enriched metabolic and oxidative phosphorylation genes, their precise role in cartilage resorption by chondroclasts remains to be established. While transcriptome is a key determinant of the phenotype of a cell, there exists a possibility that overrepresentation of a subset of gene within a cell type regulates the dynamic function of cells. However, use of gene expression as a cell marker and physiological dynamics of cell (chondroclasts) requires further characterization at single cell level using single-cell RNA sequencing studies at high depth of coverage. The broad category of core genes enriched as chondroclast signature genes may further reflect the highly dynamic, energetic, and active state of cell type studied. The homogenous in vitro culture of chondroclasts in dynamic vs static phase may further help in narrowing down and specifying the signature genes. Future studies will interrogate the functionality of these genomic changes at the cellular and tissue levels.

## Conclusions

While our comparative analysis identifies profound transcriptomic differences between two resorbing cells which specifically differ in their surrounding substrates and geographical location, it does not address the certain heterogeneity that exists at the single cell level in each location. However, given the limited range of detection of microarray-based approach, many of the differences we have identified might not have been evident with RNA sequencing approach. Although hybridization-based microarray approaches are high throughput and relatively inexpensive, they posed some limitations including high background levels and saturation signals, limited dynamic range of detection, and complex normalization methods. Nevertheless, the results presented here provide a foundation for further work to establish the cellular heterogeneity and characterize the molecular denominators of chondroclast function in vivo. Taken together, the comparative gene expression profiles provided here would be useful resources for future work to uncover novel mechanisms of bioenergetic and metabolic facet of chondroclast cells.

## Supplementary information

**Additional file 1 : Supplementary Figure 1.** Quality of RNA isolated from LCM captured TRAP positive chondroclasts and osteoclasts as assessed by Agilent Bioanalyzer. The average size of the RNA fragments was between 100 to 150 bp maxing out at around 2 kb fragments. The majority though was within the 150 nt. **Supplemental Figure 2.** Immunofluorescent staining of differentially expressed genes showing co-localization of expression in TRAP-positive cells. Nucleus staining was performed to visualize the nucleus, whereas osteoclasts and chondroclasts were shown by TRAP staining. The immunostaining was performed using validated antibodies against Lmp2, Psat1, Nxn, Ufsp2 and Wnt5a. **Supplementary Figure 3.** Topological properties of PPI network of genes up-regulated in chondroclasts. Network analysis of PPI network using several topological features including (**A**) Betweenness Centrality (**B**) Topological Coefficients (**C**) Neighborhood Connectivity Distribution and (**D**) Closeness Centrality**.**

## Data Availability

The datasets analyzed during the current study are available from the corresponding author on reasonable request.

## Abbreviations

TRAP: Tartrate-resistant acid phosphatase
KEGG: The Kyoto Encyclopedia of Genes and Genomes
MCODE: Molecular Complex Detection Algorithm
LCM: Laser capture microdissection
TF: Transcription factor
GO: Gene Ontology
BP: Biological process
MF: Molecular function
PCA: Principal component analysis
NES: Normalized enrichment score
FDR: False discovery rate
PPI: Protein-protein interaction
STRING: Search Tool for the Retrieval of Interacting Genes
DEGs: Differentially expressed genes
RANK: Receptor Activator of NF-κB
MMP: Matrix metalloproteinases
LogFC: Log fold change
