# Supplementary material for: Comparative transcriptomic analysis identifies distinct molecular signatures and regulatory networks of chondroclasts and osteoclasts
Source: Arthritis Res Ther. 2020 Jul 10;22:168. doi: 10.1186/s13075-020-02259-z (PMC7353397; doi:10.1186/s13075-020-02259-z)
Supplement: Supplementary file 1 — Additional file 1 : Supplementary Figure 1. Quality of RNA isolated from LCM captured TRAP positive chondroclasts and osteoclasts as assessed by Agilent Bioanalyzer. The average size of the RNA fragments was between 100 to 150 bp maxing out at around 2 kb fragments. The majority though was within the 150 nt. Supplemental Figure 2. Immunofluorescent staining of differentially expressed genes showing co-localization of expression in TRAP-positive cells. Nucleus staining was performed to visualize the nucleus, whereas osteoclasts and chondroclasts were shown by TRAP staining. The immunostaining was performed using validated antibodies against Lmp2, Psat1, Nxn, Ufsp2 and Wnt5a. Supplementary Figure 3. Topological properties of PPI network of genes up-regulated in chondroclasts. Network analysis of PPI network using several topological features including (A) Betweenness Centrality (B) Topological Coefficients (C) Neighborhood Connectivity Distribution and (D) Closeness Centrality. [file 13075_2020_2259_MOESM1_ESM.pptx]

## Slide 1
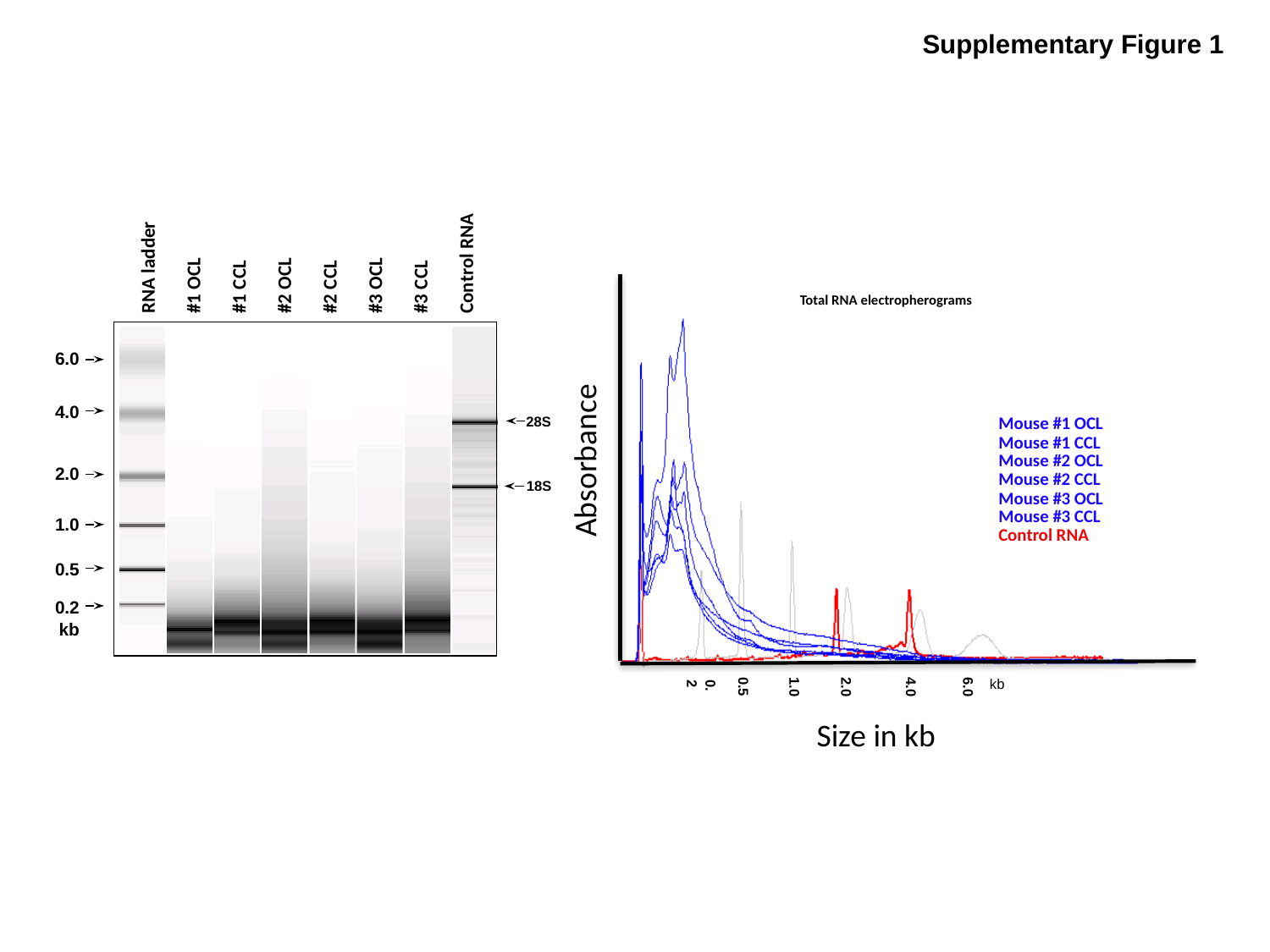

Supplementary Figure 1
RNA ladder
#1 OCL
#1 CCL
#2 OCL
#2 CCL
#3 OCL
#3 CCL
Control RNA
 Total RNA electropherograms
6.0
4.0
2.0
1.0
0.5
0.2
kb
28S
18S
Mouse #1 OCL
Mouse #1 CCL
Mouse #2 OCL
Mouse #2 CCL
Mouse #3 OCL
Mouse #3 CCL
Control RNA
Absorbance
0.2
kb
0.5
1.0
2.0
4.0
6.0
Size in kb

## Slide 2
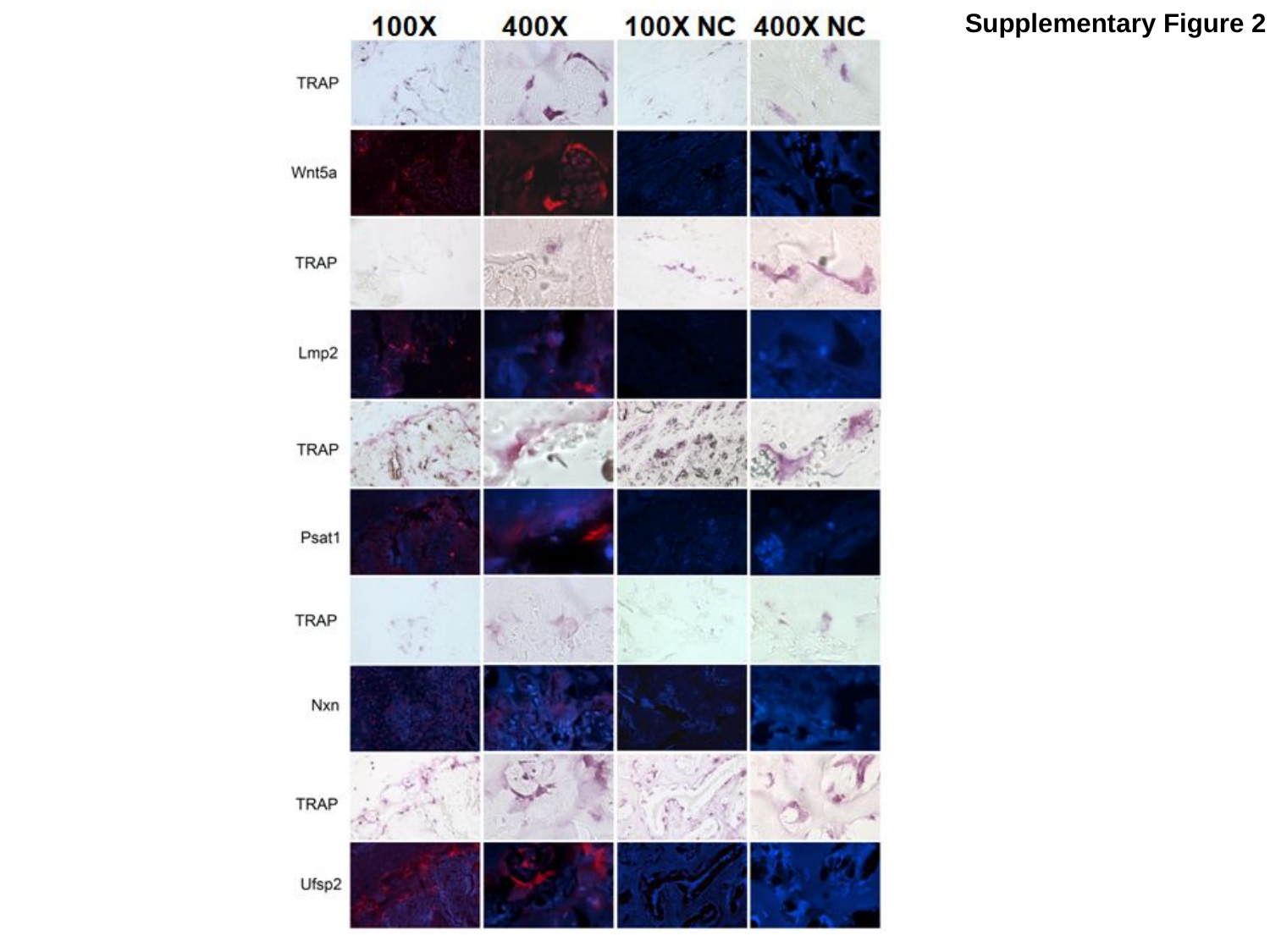

Supplementary Figure 2

## Slide 3
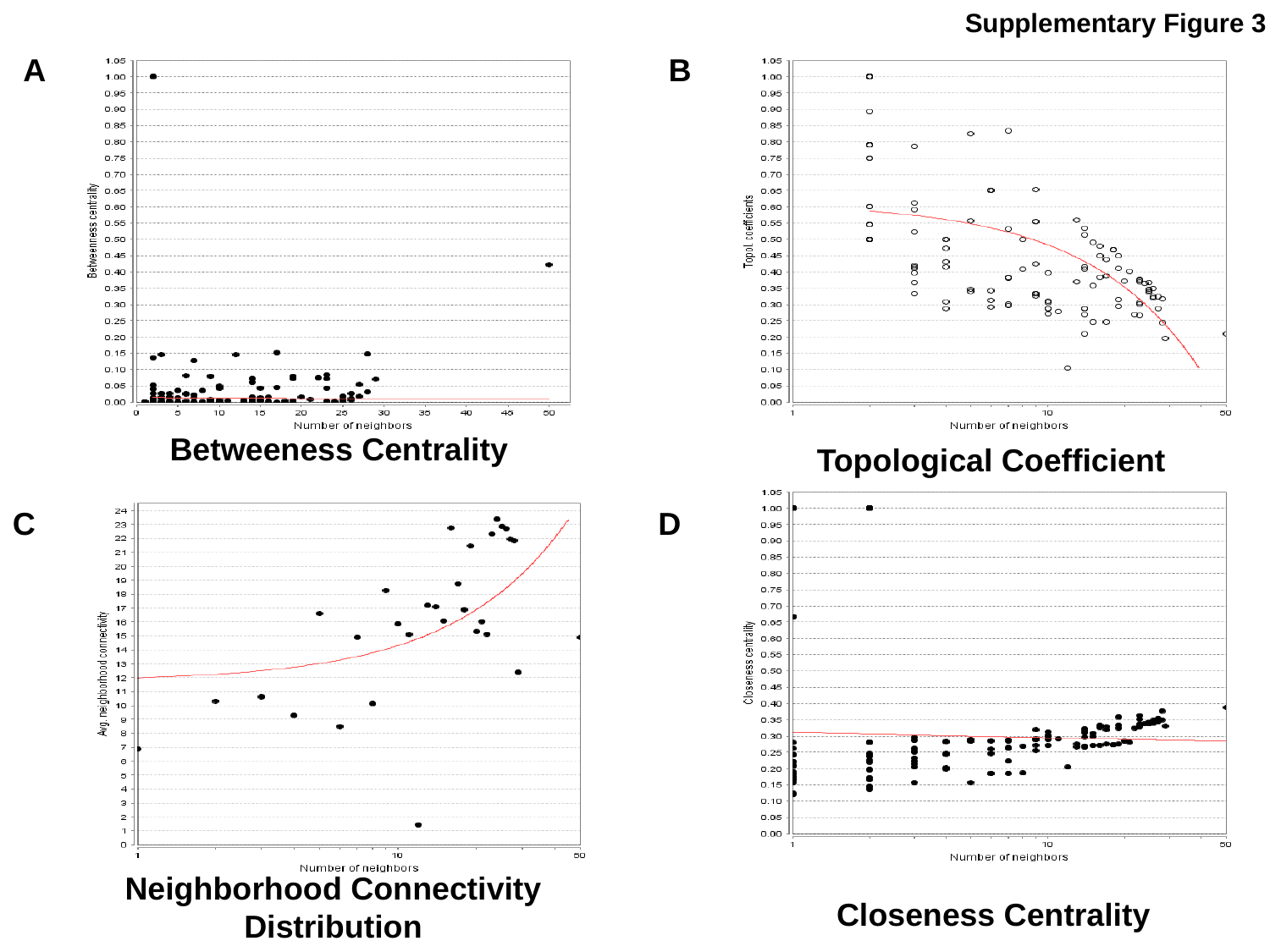

Supplementary Figure 3
A
B
Betweeness Centrality
Topological Coefficient
C
D
Neighborhood Connectivity Distribution
Closeness Centrality
